# DNA Barcoding of Two Thymelaeaceae Species: *Daphne mucronata* Royle and *Thymelaea hirsuta* (L.) Endl

**DOI:** 10.3390/plants10102199

**Published:** 2021-10-16

**Authors:** Almuthanna K. Alkaraki, Maisam A. Aldmoor, Jamil N. Lahham, Mohammed Awad

**Affiliations:** 1 Department of Biological Sciences, Faculty of Science, Yarmouk University, Irbid 21163, Jordan; 2018103017@ses.yu.edu.jo (M.A.A.); jamil@yu.edu.jo (J.N.L.); 2 Department of Biotechnology, Faculty of Agriculture, Al-Azhar University, Cairo 11651, Egypt; biotechnology_99@yahoo.com

**Keywords:** DNA barcoding, *Thymelaeaceae* family, *Daphne mucronata*, *Thymelaea hirsuta*, *matK*, *rbcL*, *rpoC1*

## Abstract

*Daphne mucronata* Royle and *Thymelaea hirsuta* (L.) Endl both belong to the *Thymelaeaceae* family. Both species are used traditionally to treat several diseases along with various daily applications by Jordanian Bedouins. Traditionally, those species are identified through personal proficiency, which could be misleading due to human errors or lack of expertise. This study aims to investigate an effective DNA barcoding method to identify and characterize *Daphne mucronata* Royle and *Thymelaea hirsuta* plant species at the molecular level. *Daphne mucronata* Royle and *Thymelaea hirsuta* were collected from the ancient city of Petra in the Southern part of Jordan. Sequences of candidate DNA barcodes were amplified (*rbcL*, *matK*, and *rpoC1*), sequenced, and aligned to the blastn database. Moreover, the obtained sequences were compared with available sequences of related species at the GenBank database. Our results showed that DNA barcoding successfully identifies the two plant species using any of chloroplast genes (*rbcL*, *matK*, or *rpoC1*). The results emphasize the ability of DNA barcoding for identifying and characterizing different plant species through the recruitment of different barcode loci in molecular identification.

## 1. Introduction

*Thymelaeaceae* family is a medium-sized family of Angiosperms that contains almost 898 species distributed in 50 different genera [1]. *Daphne* and *Thymelaea* genera comprise 95 and 30 species, respectively, representing around 23 percent of the family [2]. *Thymelaeaceae* family is widely used in folk medicine to treat several diseases as it has anti-leukemia, antitumor, anti-gout, anti-inflammatory, and antimicrobial pharmacological properties [3]. Among the *Thymelaeaceae* species are *Daphne mucronata* and *Thymelaea hirsute,* with various medical and daily uses. 

The *Daphne mucronata* Royle [4] is a wild evergreen shrub distributed in Southeast Asia, Afghanistan, Pakistan, Iran, North Africa, and South Europe [5]. *Daphne mucronata* is used in folk medicine to treat cancer, different skin disorders, ulcer, and purgative abortifacient [3,6,7,8,9]. Moreover, *Daphne mucronata* has analgesic, anti-inflammatory, and antimicrobial activities [10]. Recently, *Daphne mucronata* Royle showed a protective and anti-inflammatory effect on the stressed human adipose-derived mesenchymal stem cells protecting human adipose stem cells against monosodium iodoacetate and enhancing cell proliferation [11]. The phytochemical screening of *Daphne mucronata* Royle showed antimicrobial activity and antioxidant properties [12,13,14,15]. Moreover, ethyl acetate extract of *Daphne mucronata* aerial parts revealed the following chemical constituents: Coumarins, flavonoids, triterpenoids, diterpenes, lignin, and glucosides [10].

*Thymelaea hirsuta* (shaggy sparrow-wort or Mitnan in Arabic) is a xerophyte shrub that can grow up to two meters in height with a root system reaching up to 3.5 m depth, and is known for its fleshy tiny size leaves and flowers [16]. *Thymelaea hirsuta* is a toxic plant with reported therapeutic properties [16]. Traditionally, the leaves of *Thymelaea hirsuta* were used to treat pinworms and skin conditions in the thirteenth century, while the bark was recruited to promote wound healing [16]. In addition, local Bedouins used the inner bark of *Thymelaea hirsuta* in manufacturing ropes and paper sheets [17,18]. Additionally, Bedouins have recruited powdered *Thymelaea hirsuta* in their traditional veterinary medicine to prevent miscarriages in she-camels [17]. Generally, steroidal compounds, flavonoids, coumarins, and lignans are the active chemical constituents that play a role in biological activity [19]. The *Thymelaea* *hirsuta* aqueous extracts are highly active sources of natural antioxidants, which play an essential role in controlling various pathological conditions, such as Parkinson’s disease and Alzheimer’s disease [20]. In addition, *Thymelaea* *hirsuta* plants’ aqueous extracts are rich in polyphenol contents that show antihypertensive and antidiabetic activities, thus the plant may be considered a food supplement for diabetic and hypertensive patients [21]. Furthermore, ethanolic extracts of *Thymelaea* *hirsuta* can significantly inhibit human adenocarcinoma cell growth [22]. Many *Thymelaea* *hirsuta* extract revealed antimicrobial and antifungal activities, and exhibited an excellent antioxidant activity [23]. Phytochemical screening of *Thymelaea hirsuta* aerial parts showed the presence of alkaloids tannins, saponins, steroids, coumarins, and anthraquinones [20]. Moreover, the aqueous extract of *Thymelaea hirsuta* revealed both hypoglycaemic and antidiabetic effects in normal glycaemic and induced diabetic rats, indicating the basis for *Thymelaea hirsuta* in diabetes treatment in Folk medicine [24]. In addition to the antidiabetic effect of *Thymelaea hirsuta L*. in a rat model, an antihypertensive effect was also reported [21]. In addition, *Thymelaea hirsuta* exhibited significant activity in acute inflammation compared to a standard anti-inflammatory drug (diclofenac) [25]. A recent study highlights the traditional usage of *Thymelaea hirsuta* extracts on cutaneous dermatophytosis and the new potential use of *Thymelaea hirsuta* as antiaging and better healing of the skin [26].

*Daphne mucronata* and *Thymelaea hirsuta* are essential as herbal medicine in folk remedies and traditional applications related to the daily life of Bedouins. The importance of both species inspires the research group to establish an effective DNA barcode to distinguish both species at the molecular level. 

DNA barcoding is an identification tool of different samples based on the molecular marker of conserved regions [27,28]. DNA Barcoding is widely used to identify and classify animal and plant species; unknown samples even previously described [29,30]. Moreover, DNA barcoding is used for quality control and identification of food authentication, for example, seafood, herbal plants, and crops [31,32]. This study aims to use DNA barcoding to confirm the identity of the following two medicinal plant species: *Daphne mucronata* and *Thymelaea hirsuta* using *matK*, *rbcL*, and *rpoC1* genes as a barcode region.

## 2. Results

**Figure 1:**
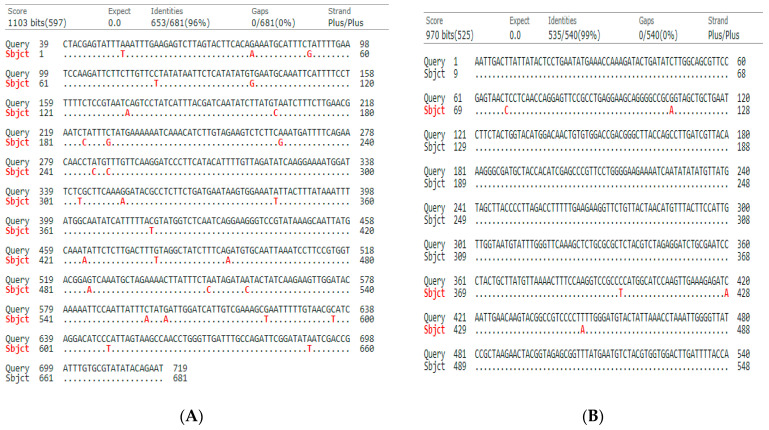
Pairwise alignment of *Daphne mucronata* and *Thymelaea hirsuta* using dots method (BLAST): (**A**) *matK* of *Daphne mucronata* (Query) and *Thymelaea hirsuta* (subject); (**B**) *rbcL* of *Daphne mucronata* (Query) and *Thymelaea hirsuta* (subject).

DNA was isolated, and targeted sequences were amplified using the selected PCR primers for the four barcode loci of *Daphne mucronata* and *Thymelaea hirsuta* (L.) Endl. DNA sequencing was successfully performed for 5 out of 6 loci in both selected plant species (Table 1). *Daphne mucronata* and *Thymelaea hirsuta* selected barcode regions were searched against the GenBank database [33]. Obtained sequences (Appendix A) were deposited at the GenBank database [33], and the deposited accession numbers are shown in Table 1. Barcode sequences were not retrieved for *Daphne mucronata* for the four selected barcode loci, while *Thymelaea hirsuta* retrieved sequences for only *matK and rbcL* (see retrieved accessions in Table 1). The obtained barcode sequences for *matK* and *rbcL* showed 97.96% identity for *matK* and 100% for *rbcL* of the retrieved two accessions of *Thymelaea hirsute*. The obtained sequences of both species were aligned using a pairwise alignment search tool (Blastn). The two plant species showed 96% of identity for *matK*, and 99% for *rbcL,* as shown in Figure 1.

The obtained sequences were run in blastn, and five high match scores were chosen to run phylogenetic analysis. The five related sequences were selected according to the highest BLAST hits. The retrieved genes of different species related to *Daphne mucronata* and *Thymelaea hirsute*, along with E values, identity percentage, and the retrieved accessions, are shown in Table 2. Unavailable sequences (specific genes) for selected species was obtained by extracting the selected genes from the complete chloroplast genome via python code.

The results show that the percentage identity range was the highest (99.16%) between *Daphne mucronata matK*, and both *Daphne longilobata* and *Daphne tangutica*. In comparison, the lowest percentage of identity was reported in *Daphne mucronata matK* barcode locus (98.04%) and *Daphne giraldii* species, belonging to the *Thymelaeaceae* family. The highest identity percentage was among *Thymelaea hirsuta rbcL* (100.00%) reported earlier in the database, followed by 99.26% found in *Daphne mezereum rbcL, Stellera chamaejasme rbcL,* and *Wikstroemia monnula rbcL* (Table 2). 

**Figure 2:**
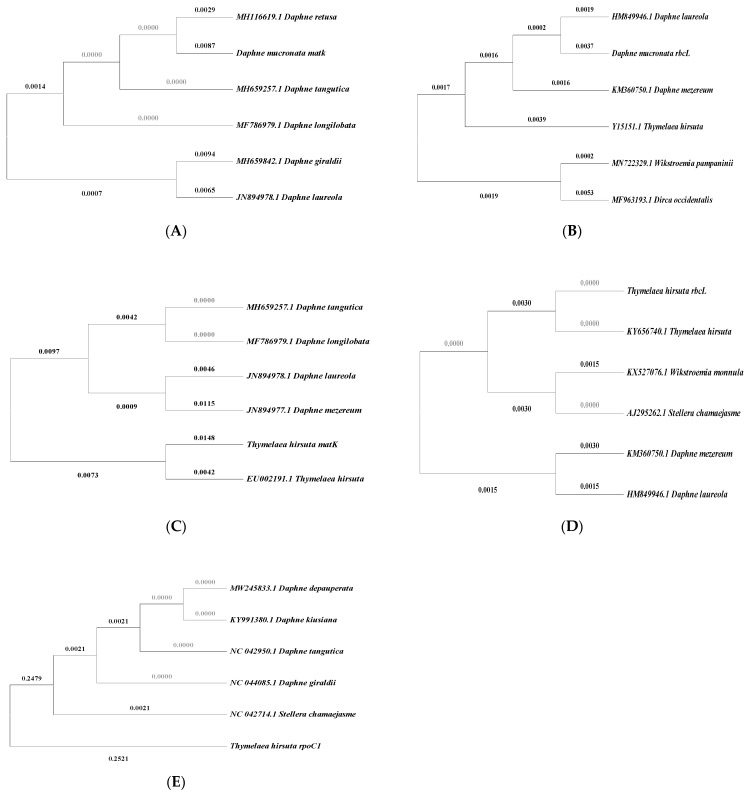
The phylogenetic trees (Neighbor-Joining method) of the top five related species and obtained barcode sequences of *Thymelaea hirsuta* and *Daphne mucronata*. (**A**) *Daphne mucronata matK*, the sum of branch length is 0.02958153; (**B**) *Daphne mucronata rbcL*, the sum of branch length is 0.02199074; (**C**) *Thymelaea hirsuta matK*, the sum of branch length is 0.05720029; (**D**) *Thymelaea hirsuta rbcL*, the sum of branch length is 0.01331361; (**E**) *Thymelaea hirsuta rpoC1*, the sum of branch length is 0.50635593.

The top five related sequences that appeared in Table 2 were recruited in phylogenetic trees construction using Mega X software shown in (Figure 2). Figure 2 shows phylogenetic trees of *Daphne mucronata* related species using *matK,* and *rbcL* barcode loci. The *matK* barcode could discriminate *Daphne mucronata* from other related species (Figure 2A), while *rbcL* can discriminate between *Daphne mucronata* and *Daphne mezereum, Daphne laureola, Dirca occidentalis,* and *Thymelaea hirsute* (Figure 2B). In Figure 2, phylogenetic trees of *Thymelaea hirsuta* and other related species show that *matK* can discriminate between *Thymelaea hirsuta, Daphne laureola,* and *Daphne mezereum (matK, rbcL,* and *rpoC1*) barcode loci (Figure 2C). While Figure 2D shows that *rbcL* can discriminate between *Thymelaea hirsuta* and the five related species. The *rpoC1* can discriminate between *Thymelaea hirsuta* and *Stellera chamaejasme (*Figure 2E). Further analysis was performed through the NCBI-Taxonomy browser to check the ability of the obtained sequences to fit within the proper plant family (*Thymelaeaceae*). Table 3 shows the number of obtained hits (organisms) according to the taxonomy browser (NCBI), once running sequences through blastn (NCBI) database. In Table 3 the NCBI taxonomy Entrez results of the retrieved lineage hits support that all sequences are be able to be discriminated and retained to *Thymelaeaceae* family. 

## 3. Discussion

Jordanian Flora is rich with an enormous variety of plant species belonging to 112 plant families, where more than 363 species are considered medicinal due to their therapeutic activity [34,35,36]. In Jordan, the *Thymelaeaceae* family is represented by two genera *Daphne* (*Daphne mucronata* Royle) and *Thmelaea* (three species; *Thymelaea hirsuta, Thymelaea passerine, and Thymelaea pubescens*) [37]. *Daphne mucronata* is distributed in Petra, Karak, Ma’an, and Tafila [38]. At the same time, *Thymelaea hirsuta* is distributed in the southern part of Jordan (Petra, Tafila, Shobak, and Ma’an) [37,38]. The usage of both selected species in folk medicine and the recruitment of *Thymelaea hirsuta* in Bedouins’ daily life makes both species excellent candidates for molecular identification (barcoding). 

Much research was conducted to investigate the therapeutic and antioxidant activities of both *Daphne mucronata* and *Thymelaea hirsute*. However, molecular identification and phylogenetic characterization were very limited. Exploring the GenBank database for *Daphne mucronata* retrieved no results [33], indicating that our obtained sequences are new and firsthand. At the same time, *Thymelaea hirsuta* search retrieved deposited sequences for both *rbcL* and *matK* sequences but nothing for both *rpoC1* [39]. The length of gene sequences is within the average length, satisfying the previously reported criteria [40]. In addition, DNA barcoding was successfully identified *Thymelaea hirsuta* and *Daphne mucronata* species. A total of 5 sequences were successfully obtained for the two plant species using different chloroplast barcode loci (*rbcL, matK,* and *rpoC1*). Among those sequences, about 3 novel sequences were not included earlier within the GenBank database (OK188786, OK040775, OK040776). Moreover, the identity percent between our *Thymelaea hirsuta* sequence and previously deposited sequence in GenBank database is 97.96% for *matK* and 100.00% for *rbcL*. 

The Molecular phylogenetic relationships of different species from *Thymelaeaceae* family sequences from Africa and Australia were investigated earlier by parsimony analysis [41], including *Thymelaea hirsuta* Endl (the original sequence was obtained from [42]). The van der Bank study was limited to *rbcL*, *trnL intron*, and *trnL-F* intergenic spacer sequences, and separate sequence analysis of the selected sequences produced nonidentical phylogenetic outcomes. Meanwhile, combined sequences analysis did improve the resolution of phylogenetic discrimination among different clades [41]. Furthermore, *Daphne mucronata* sequences were not included in the study mentioned above [41]. In another recent study, phylogenetic analysis using maximum parsimony and Bayesian inference of the internal transcribed spacer (*ITS*) and *rbcL*, *trnL* intron, and *trnL-F* intergenic spacer revealed that the *Thymelaeaceae* is not a monophyletic family [43]. The discrimination capacity of *matK*, *rbcL*, and *rpoC1* barcode regions were divergent among studied species, indicating that each species could recruit different locus (loci), in terms of identification and molecular characterization. However, the discrimination capacity of *rpoC1* as a candidate barcode region is limited and needs future study. Lower discrimination capacity of *rpoC1* compared with *matK* and *rbcL* is probably due to limited sequences availability in reference databases for *rpoC1*, which lead to low identification capacity [44]. Many studies in plant DNA barcoding used *matK* and *rbcL* genes as barcode regions. Further studies should be done using other barcode genes, as there is no universal primer found effective in plants. DNA barcoding can be used to identify plant species, specifically medicinal plants. Further research should be carried out to establish a complete DNA barcodes database of all medicinal plants.

## 4. Materials and Methods

Fresh leaves of the two selected species from the *Thymelaeaceae* family (*Daphne mucronata* and *Thymelaea hirsuta* (L.) Endl) were collected from the ancient city of Petra (Jordan) (Locality: 30.324181945297152, 35.47997922146477). Samples collection was conducted via a specilized plant taxonomist [37]. Stored leaves were ground using liquid nitrogen, and DNA was extracted using commercial kits (Qiagen). DNA quality and quantity were checked spectrophotometrically and via 1% gel electrophoresis before the PCR amplification. Different Chloroplast loci (*matK*, *rbcL,* and *rpoC1*) were amplified using the following primers: matK (Forward—CCCRTYCATCTGGAAATCTTGGTTC and reverse—GCTRTRATAATGAGAAAGATTTCTGC) [45], *rbcL* (Forward—TGTCACCACAAACAGAAAC and reverse—TCGCATGTACCTGCAGTAGC) [46], and *rpoC1* (—GGCAAAGAGGGAAGATTTCG and reverse—CCATAAGCATATCTTGAGTTGG) [47]. PCR amplifications were conducted using 5× HOT FIREPol^®^ Blend master mix; Initial denaturation (5 min, 95 °C), followed by 40 cycles of denaturation (30 s, 95 °C), annealing (30 s at 54 °C). The final extension cycle (30 s at 72 °C) was applied for all PCR reactions, and amplified DNA fragments were qualitatively checked via Agarose gel electrophoresis before sequencing. The Amplified fragments were purified and sequenced using Sanger sequencing method (ABI PRISM^®^ kit, Macrogen company, Korea). Chromatograms were analyzed using FinchTV software [48], and obtained sequences were further analyzed using the NCBI-BLAST online tool [49] to check related sequences in the nucleotide database. Furthermore, five related sequences with a high matching score were obtained from NCBI-GenBank Entrez for further phylogenetic analysis for each plant sample. Corresponding genes were extracted using python code for species with complete chloroplast genomes [50]. Neighbor-joining phylogenetic trees were constructed using MEGA X software [51] to evaluate the phylogenetic relationships and the effectiveness of barcode discrimination at the species level. Obtained sequences were further analyzed using the NCBI taxonomy database (Lineage), via counting the number of (hits) organisms along appeared in taxonmy browser, once running the obtained sequences through NCBI blastn.

## Figures and Tables

**Table 1:** The length of *matK, rbcL,* and *rpoC1* barcode sequences in *Daphne mucronata and Thymelaea hirsute***,** along with the list of available sequences of *Daphne mucronata* and *Thymelaea hirsuta* that were retrieved from the GenBank database and our deposited sequences at GenBank [33].

Plant Species	Sequences Length (bp)		
	*matK*	*rbcL*	*rpoC1*
*Daphne mucronata*	724	540	-*
Available GenBank accession number	N/A **	N/A	N/A
Deposited accession number at GenBank	MZ851783	OK188786	-
*Thymelaea hirsuta*	685	682	479
Available GenBank accession number	EU002191.1	KY656740.1	N/A
Deposited accession number at GenBank	OK040774	OK040775	OK040776

* Unspecific amplification was obtained; ** N/A Unavailable at GenBank database.

**Table 2:** The NCBI-BLAST results retrieved sequences of different species related to Daphne mucronata, sequence coverage (QC), E value, identity percentage, and retrieved accessions.

Plant Species	Gene	Related Species	QC	E-Value	Identity	Accession
*Daphne mucronata*	*matk*	*Daphne longilobata*	98%	0	99.16%	MF786979.1
	*matk*	*Daphne tangutica*	98%	0	99.16%	MH659257.1
	*matk*	*Daphne laureola*	99%	0	98.33%	JN894978.1
	*matk*	*Daphne retusa*	95%	0	98.85%	MH116619.1
	*matk*	*Daphne giraldii*	98%	0	98.04%	MH659842.1
*Daphne mucronata*	*rbcL*	*Daphne mezereum*	100%	0	99.44%	KM360750.1
	*rbcL*	*Daphne laureola*	100%	0	99.44%	HM849946.1
	*rbcL*	*Thymelaea hirsuta*	100%	0	99.07%	Y15151.1
	*rbcL*	*Wikstroemia pampaninii*	100%	0	99.07%	MN722329.1
	*rbcL*	*Dirca occidentalis*	100%	0	98.52%	MF963193.1
*Thymelaea hirsuta*	*matk*	*Thymelaea hirsuta*	100%	0	97.96%	EU002191.1
	*matk*	*Daphne laureola*	100%	0	96.21%	JN894952.1
	*matk*	*Daphne tangutica*	100%	0	96.36%	MH659257.1
	*matk*	*Daphne longilobata*	100%	0	96.36%	MF786979.1
	*matk*	*Daphne mezereum*	100%	0	95.77%	JN894977.1
*Thymelaea hirsuta*	*rbcL*	*Thymelaea hirsuta*	99%	0	100.00%	KY656740.1
	*rbcL*	*Daphne laureola*	99%	0	99.41%	HM849946.1
	*rbcL*	*Daphne mezereum*	99%	0	99.62%	KM360750.1
	*rbcL*	*Stellera chamaejasme*	99%	0	99.62%	AJ295262.1
	*rbcL*	*Wikstroemia monnula*	99%	0	99.62%	KX527076.1
*Thymelaea hirsuta*	*rpoC1* *	*Daphne giraldii*	97%	0	99.15%	NC_044085.1
	*rpoC1* *	*Daphne tangutica*	97%	0	99.15%	NC_042950.1
	*rpoC1* *	*Stellera chamaejasme*	97%	0	99.15%	NC_042714.1
	*rpoC1* *	*Daphne kiusiana*	97%	0	99.15%	KY991380.1
	*rpoC1* *	*Daphne depauperate*	97%	0	99.15%	MW245833.1

* Complete genome of chloroplast was found with an accession number then genes extracted by Python code.

**Table 3:** NCBI taxonomy Entrez results; running obtained sequences via blastn and retrieving the lineage hits and number of aligned sequences related to *Thymelaeaceae* family.

Sequence (Organism)	Taxonomy	Number of Hits	Number of Organisms
*matK (Daphne Mucronata)*	*Thymelaeaceae*	104	32
*rbcL (Daphne Mucronata)*	*Thymelaeaceae*	119	66
*matK (Thymelaea hirsuta)*	*Thymelaeaceae*	105	32
*rbcL (Thymelaea hirsuta)*	*Thymelaeaceae*	118	66
*rpoC1 (Thymelaea hirsuta)*	*Thymelaeaceae*	101	43

## Appendix A

**Table A1:** List of obtained plant samples sequences in FASTA format.

**>seq1 [organism= *Daphne mucronata*] *matK* gene, partial cds, GenBank Accession Number = MZ851783.**						
GATGCCTCTT	TTTTGCATTT	ATTACGGCTT	CTTTTTTTCT	ACGAGTATTT	AAATTTGAAG	60
AGTCTTAGTA	CTTCACAGAA	ATGCATTTCT	ATTTTGAATC	CAAGATTCTT	CTTGTTCCTA	120
TATAATTCTC	ATATATGTGA	ATGCAAATTC	ATTTTCCTTT	TTCTCCGTAA	TCAGTCCTAT	180
CATTTACGAT	CAATATCTTA	TGTAATCTTT	CTTGAACGAA	TCTATTTCTA	TGAAAAAATC	240
AAACATCTTG	TAGAAGTCTC	TTCAAATGAT	TTTCAGAACA	ACCTATGTTT	GTTCAAGGAT	300
CCCTTCATAC	ATTTTGTTAG	ATATCAAGGA	AAATGGATTC	TCGCTTCAAA	GGATACGCCT	360
CTTCTGATGA	ATAAGTGGAA	ATATTACTTT	ATAAATTTAT	GGCAATATCA	TTTTTACGTA	420
TGGTCTCAAT	CAGGAAGGGT	CCGTATAAAG	CAATTATGCA	AATATTCTCT	TGACTTTGTA	480
GGCTATCTTT	CAGATGTGCA	ATTAAATCCT	TCCGTGGTAC	GGAGTCAAAT	GCTAGAAAAC	540
TTATTTCTAA	TAGATAATAC	TATCAAGAAG	TTGGATACAA	AAATTCCAAT	TATTTCTATG	600
ATTGGATCAT	TGTCGAAAGC	GAATTTTTGT	AACGCATCAG	GACATCCCAT	TAGTAAGCCA	660
ACCTGGGTTG	ATTTGCCAGA	TTCGGATATA	ATCGACCGAT	TTGTGCGTAT	ATACAGAATC	720
TTCT						
**>seq2 [organism= *Daphne mucronata*] *rbcL gene*, partial cds, GenBank Accession Number = OK188786.**						
AATTGACTTA	TTATACTCCT	GAATATGAAA	CCAAAGATAC	TGATATCTTG	GCAGCGTTCC	60
GAGTAACTCC	TCAACCAGGA	GTTCCGCCTG	AGGAAGCAGG	GGCCGCGGTA	GCTGCTGAAT	120
CTTCTACTGG	TACATGGACA	ACTGTGTGGA	CCGACGGGCT	TACCAGCCTT	GATCGTTACA	180
AAGGGCGATG	CTACCACATC	GAGCCCGTTC	CTGGGGAAGA	AAATCAATAT	ATATGTTATG	240
TAGCTTACCC	CTTAGACCTT	TTTGAAGAAG	GTTCTGTTAC	TAACATGTTT	ACTTCCATTG	300
TTGGTAATGT	ATTTGGGTTC	AAAGCTCTGC	GCGCTCTACG	TCTAGAGGAT	CTGCGAATCC	360
CTACTGCTTA	TGTTAAAACT	TTCCAAGGTC	CGCCCCATGG	CATCCAAGTT	GAAAGAGATC	420
AATTGAACAA	GTACGGCCGT	CCCCTTTTGG	GATGTACTAT	TAAACCTAAA	TTGGGGTTAT	480
CCGCTAAGAA	CTACGGTAGA	GCGGTTTATG	AATGTCTACG	TGGTGGACTT	GATTTTACCA	540
**>seq3 [organism=*Thymelaea hirsuta*] *matK* gene, partial cds, GenBank Accession Number =** **OK040774.**						
CTACGAGTAT	TTTAATTTGA	AGAGTCTTAG	TACTTCACAA	AAATGCATTT	CGATTTTGAA	60
TCCAAGATTC	TTCTTGTTCT	TATATAATTC	TCATATATGG	GAATGCAAAT	TCATTTTCCT	120
TTTTCTCCGT	AATAAGTCCT	ATCATTTACG	ATCAATATCT	TATGCAATCT	TTCTTGAACG	180
AATCCATTTG	TATGAAAAAA	TCAAACATCT	TGTAGAAGTC	TCTTCGAATG	ATTTTCAGAA	240
CAACCTCTGC	TTGTTCAAGG	ATCCCTTCAT	ACATTTTGTT	AGATATCAAG	GAAAATGGAT	300
TCTTGCTTCA	AAAGATACGC	CTCTTCTGAT	GAATAAGTGG	AAATTTTACT	TTATAAATTT	360
ATGGCAATAT	CATTTTTATG	TATGGTCTCA	ATCAGGAAGG	GTCCGTATAA	AGCAATTATG	420
CAAAAATTCT	CTTGACTTTT	TAGGCTATCT	TTCAAATGTG	CAATTAAATC	CTTCCGTGGT	480
ACGGAATCAA	ATGCTAGAAA	ACTTATTTCT	CATAGATACT	ACTATCAAGA	AGTTGGATAC	540
AAAAATTCCA	ATTATTTATA	TAATTGGATC	ATTGTCGAAA	GCTAATTTTT	GTAACGTATC	600
AGGACATCCT	ATTAGTAAGC	CAACCTGGGT	TGATTTGCCA	GATTCGGATA	TTATCGACCG	660
ATTTGTGCGT	ATATACAGAA	TTTTT				685
**>seq4 [organism=*Thymelaea hirsuta*] *rbcL* gene, partial cds, GenBank Accession Number =** **OK040775.**						
AGAGTATAAA	TTGACTTATT	ATACTCCTGA	ATATGAAACC	AAAGATACTG	ATATCTTGGC	60
AGCGTTCCGA	GTAACCCCTC	AACCAGGAGT	TCCGCCTGAG	GAAGCAGGGG	CCGCAGTAGC	120
TGCTGAATCT	TCTACTGGTA	CATGGACAAC	TGTGTGGACC	GACGGGCTTA	CCAGCCTTGA	180
TCGTTACAAA	GGGCGATGCT	ACCACATCGA	GCCCGTTCCT	GGGGAAGAAA	ATCAATATAT	240
ATGTTATGTA	GCTTACCCCT	TAGACCTTTT	TGAAGAAGGT	TCTGTTACTA	ACATGTTTAC	300
TTCCATTGTT	GGTAATGTAT	TTGGGTTCAA	AGCTCTGCGC	GCTCTACGTC	TAGAGGATCT	360
GCGAATCCCT	ACTGCTTATG	TTAAAACTTT	CCAAGGTCCG	CCTCATGGCA	TCCAAGTTGA	420
AAGAGATAAA	TTGAACAAGT	ACGGCCGTCC	CCTATTGGGA	TGTACTATTA	AACCTAAATT	480
GGGGTTATCC	GCTAAGAACT	ACGGTAGAGC	GGTTTATGAA	TGTCTACGTG	GTGGACTTGA	540
TTTTACCAAA	GATGATGAGA	ATGTGAACTC	CCAACCATTT	ATGCGTTGGA	GAGACCGTTT	600
CTTATTTTGT	GCCGAAGCAA	TTTATAAAGC	ACAGGCTGAA	ACAGGTGAAA	TCAAAGGGCA	660
TTACTTGAAT	GCTACTGCAG	GA				
**>seq5 [organism=*Thymelaea hirsuta*] *rpoC1* gene, partial cds, GenBank Accession Number =** **OK040776.**						
GATCATACGG	GCGTTCTGTC	ATTGTTGTTG	GCCCCTCACT	TTCATTACAT	CGCTGTGGGT	60
TGCCTCGCGA	AATAGCAATA	GAGCTTTTCC	AGACATTTGT	AATTCGCGGT	CTAATTAGAC	120
AACATCTTGC	TTCGAACATA	GGAGTTGCTA	AGAGTAAAAT	TCGCGAAAAG	GGGCCGATTG	180
TATGGCAAAT	ACTTCAAGAA	GTTATGCAGG	GGCATCCTGT	ATTGCTGAAT	AGAGCGCCTA	240
CTCTGCATAG	ATTAGGGATA	CAGGCATTCG	AGCCCATTTT	AGTGGAAGGG	CGTGCTATTT	300
GTTTACATCC	ATTGGTTTGT	AAGGGATTTA	ATGCAGACTT	TGATGGGGAT	CAAATGGCTG	360
TTCATGTACC	TTTGTCTTTA	GAGGCTCAAG	CAGAGGCTCG	TTTACTTATG	TTTTCTCATA	420
TGAATCTCTT	GTCTCCAGCT	ATTGGGGATC	CTATTTCTGT	ACCAACTCAA	GATAAGCGC	479
